# Degradation dynamics of quantum dots in white LED applications

**DOI:** 10.1038/s41598-021-02714-0

**Published:** 2021-12-17

**Authors:** Hsiao-Chien Chen, Abdul Shabir, Cher Ming Tan, Preetpal Singh, Jia-Hung Lin

**Affiliations:** 1 Center for Reliability Sciences and Technologies, Chang Gung University, Taoyuan, 333 Taiwan; 2 Kidney Research Center, Department of Nephrology, Chang Gung Memorial Hospital, Linkou, Taoyuan, 333 Taiwan; 3 Department of Electronic Engineering, College of Engineering, Chang Gung University, Taoyuan, 333 Taiwan; 4 Urology Department, Chang Gung Memorial Hospital, Taoyuan, 333 Taiwan; 5 Center for Reliability Engineering, Ming Chi University of Technology, New Taipei City, 243 Taiwan

**Keywords:** Electrical and electronic engineering, Lasers, LEDs and light sources, Optical materials and structures, Electronics, photonics and device physics

## Abstract

Quantum Dots (QDs) are being investigated in a hybrid white light LED structure which inculcates phosphor in the package with a blue LED chip as the light source recently. In this work, Zn doped CdS QD with ZnS shell together with green light emission phosphor is used. Upon prolonged operation, degradation of the LEDs due to the degradation of QDs is observed, which can limit its practical applications. The degradation includes intensity reduction as well as blue shift of the emitted wavelength from the white light. Three stages of degradation are observed, namely an enhancement state where light intensity is found to increase, followed by a rapid degradation stage where light intensity decreases rapidly, and finally a slower degradation stage where the degradation rate of light intensity slows down and continues till the end of the test. Through various detail material analysis, with confirmation from the density functional theory (DFT) calculations, we find that the degradation of the LEDs is due to the time evolving degradation of CdS core structure, beginning from the oxidation of sulfur vacancy of CdS QDs by the nearby oxygen atoms as a result of imperfection of the ZnS protective coating around the QDs in the presence of blue light. This oxidation renders a transformation of CdS into CdO at the initial stage. The final stage is the formation of CdSO_4_ via some intermediate processes.

## Introduction

The application of light-emitting diodes (LEDs) in display and lighting has been a topic of extensive research and development since the past decade^1^. Especially, the development of white light LEDs are used to replace the conventional lighting sources such as fluorescent and incandescent lamps. With the accelerated penetration of white light LEDs in the lighting field, the standards to evaluate the quality of white light LED source is increasing. The focus on white LED light sources has changed from the initial pursuit of only high brightness sources to high quality that considers both color rendering index and color temperature.

The traditional fabrications of white light LED is to combine three LED chips which emits red, green and blue primary colors respectively^2^. Phosphors that emit yellow light have also been developed to replace the mixed colors of red and green^3,4^. Since the emergence of quantum dots (QDs) technology, numerous researchers have been focused on the feasibility of replacing traditional red-light luminescent materials with QDs^5–8^. QDs exhibit the advantages of excellent luminescence properties, high quantum efficiency, adjustable emission wavelength through variation in size and narrow emission spectrum^9,10^. Generally, for the fabrication of white light QDs LED (QDLED), a blue LED is used as light source followed by a green phosphor and red QDs layer above it to produce white light. However, the large specific surface area of nanosized crystalline particles are prone to oxidation and degradation under light and heat^11,12^. These phenomenon leads to the decrease of luminous intensity and the shift of emission wavelength. Although the mechanisms of oxidation and degradation of QDs had been reported individually^13,14^, the performance and durability of their integration into QDLED package associated with these degradation mechanisms under ambient moisture and temperature is yet to be studied.

In this work, a packaged LED structure is fabricated with blue LED as a light source and QDs- phosphor layers as the down conversion materials. While, more attention is focussed towards the QDs characteristics and its impact on the overall LED light output as phosphor degradation is already a well-studied material in packaged LEDs^15^. Therefore, this work analyzes the QDs degradation behavior systematically and its impact on the overall light output when it is used in a hybrid structure with phosphor to produce white light. Various unique light output degradation trends are observed, and investigations of their causes are performed for better understanding of these behaviors. Furthermore, the spectroscopic methods are used to identify the overall QDs degradation over the test duration. And finally, these hypotheses of chemical failure analysis of QDs from the spectroscopic information was be further confirmed by *ab-initio* density functional theory (DFT) simulations which are an excellent tool to predict energy of formation, transition states, band structures, etc. Thus, we conceive and report a novel idea of LED failure analysis in this paper using both experimental and simulation tools to identify failure mechanisms with higher accuracy.

## Experimentation and analysis methods

**Figure 1:**
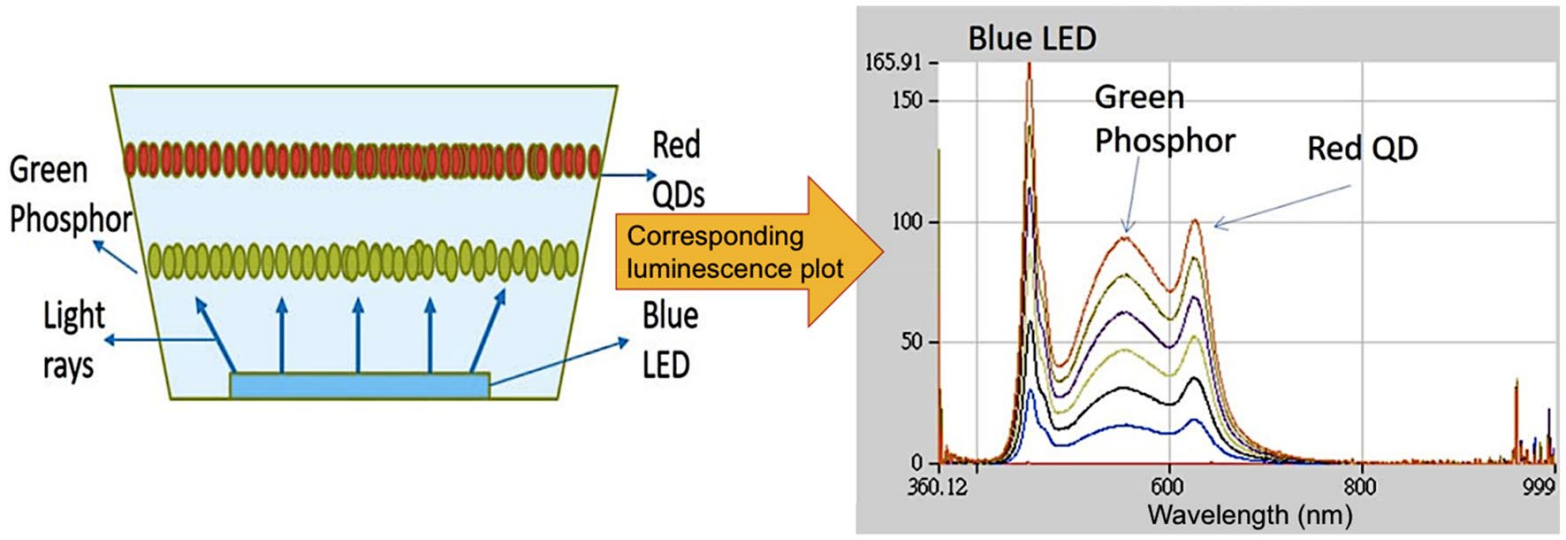
Schematic of LED structure and its corresponding luminescence plot.

5 LED samples are prepared using blue LED chips as their illumination sources. The schematic of the LEDs structure and its corresponding luminescence plot is shown in Fig. 1, which is constructed by blue LED, phosphor for emitting green light and Zn doped CdS/ZnS QDs for emitting red light. In which, the red band emission is endorsed to the sulfur vacancies and Zn dopant^12,13^. The three parts of structure contribute a wide emitting spectroscopy and result in a white light emission (Fig. 1). The fabrication of the QD is described in the supporting information S.1, and the phosphor (yttrium aluminum garnet, YAG) is green light emission purchased from Grirem Advanced Materials Co., Ltd. The InGaN/GaN blue LED chip is from Epistar Corporation.

To investigate the stability of these LED devices, experimental duration of the LEDs operating under ambient temperature of 295.8 K and relative humidity of approximately 44% RH is set to 900 h unless a 20% of lumen decay is detected earlier. The details of the degradation is described in the next section.

An initial set of measurements for all the samples are done to serve as reference baseline for each test sample. These measurements include electrical and lumen measurements. Lumen measurement is done using Mentor’s TeraLED integrating sphere and Electrical measurement is done using Keithley 2461 Source meter. The blue LED in each sample is powered with 30-mA constant current by Keithley power supply model 2651A. The optical parameters, namely light intensity and peak wavelength are monitored every 3 h. LED temperature was performed before and after the test in room temperature using IR Camera (CHCT P384A-20 Thermal Scanning System).

QDs emission parameters such as peak wavelength and intensity are studied as peak wavelength changes can result from a change in the size of the nanoparticles^14,15^. In this work, more attention is given towards the QDs characteristics and its impact on the overall LED light output as phosphor is already a well-studied material in packaged LEDs.

To investigate the dynamics of the degradation, material analysis using energy dispersive spectroscopy (EDS) and Raman spectroscopy are performed. Raman analysis showing valuable insights on the hypotheses of chemical transformations of the QDs reacting with different reagents over time is also employed. These hypotheses are further confirmed by ab-initio density functional theory (DFT) simulations which is an excellent tool to predict energy of formation, transition states, band structures, etc. This approach of investigating degradation dynamics using both experimental and simulation tools to identify failure mechanisms with increased accuracy is a novel approach.

The detail of the DFT simulation is described in the supporting information S.2, and the detail of the measurement can be found in the supporting information S.3.

## Results and discussion

### Performance of LEDs with operation time

**Figure 2:**
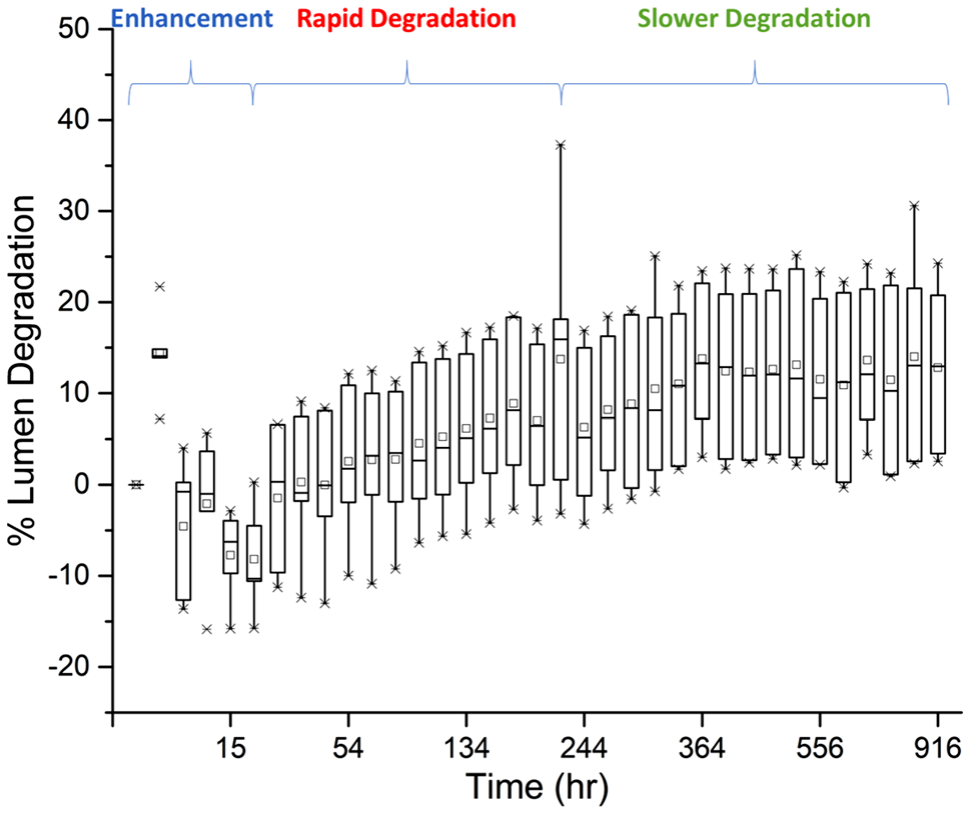
Overall LED luminescence variation with time (The whiskers indicate the outlier points in each dataset. The line in the middle of the box represents the value of median range for each dataset).

The degree of lumen degradation over test time is shown in Fig. 2, and the overall luminescence intensity weaken with test time, revealing degradation occurs in the duration of working condition.

According to the lumen variation shown in Fig. 2, the degradation can be divided into three stages, namely enhancement stage, rapid degradation stage and slower degradation stage, respectively. Stage 1 is termed as enhancement state where light intensity is observed to increase for the first 18 h of test. Stage 2 is termed as rapid degradation stage where light intensity decreases rapidly from 18 to 220 h of test. Stage 3 is termed as slower degradation stage or stable stage where light intensity degradation rate slows down. This stage continues till the end of the test of 900 h and the light intensity degrades to around 80% of its initial value. Each stage has different characteristics and hence they are likely to have different causes and implications on the overall reliability and efficiency of the structure.

**Figure 3:**
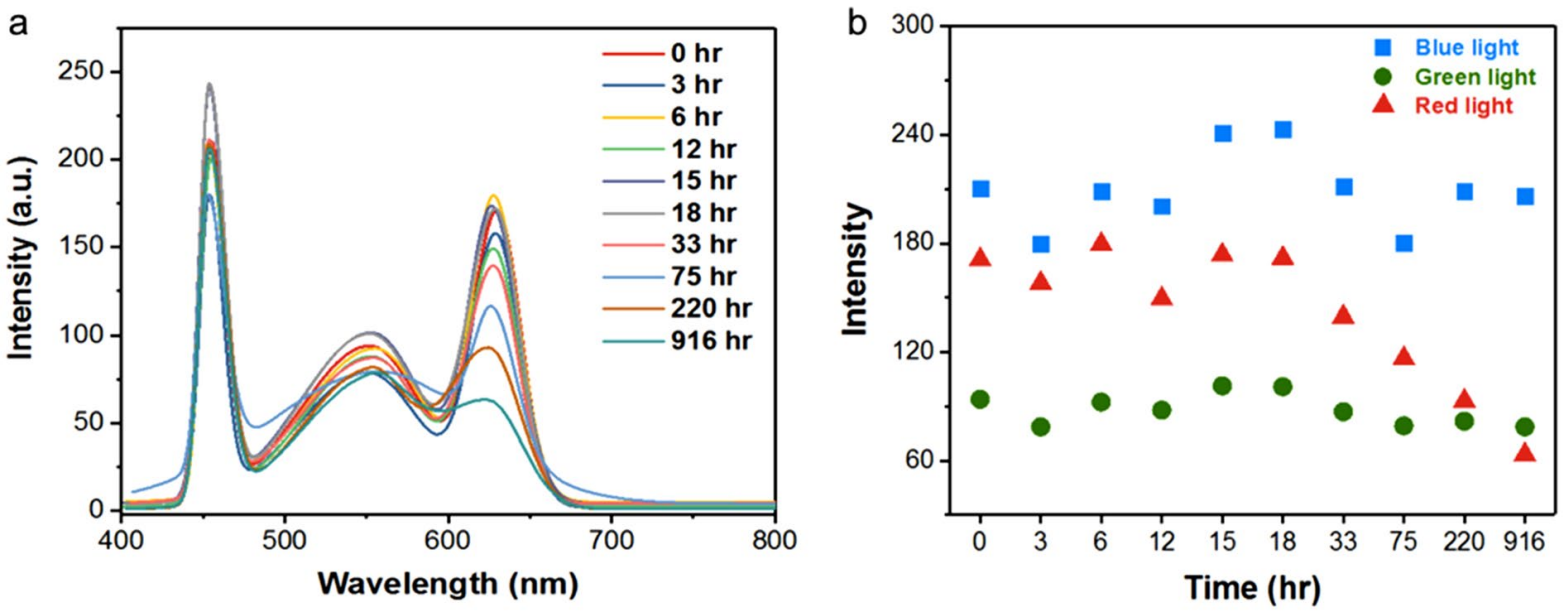
(**a**) Overall packaged LED’s luminescence data. (**b**) The variations of maximum emission intensity of blue, green and red lights.

Lumen degradation can be due to the degradation of either blue LED, phosphor, and QDs or the combination of either of these. To clarify the sources of the degradation behaviors, light intensity variations with respect to its wavelengths are separated as shown Fig. 3. The maximum intensity of blue, green and red light emission are summarized in Fig. 3b. It can be observed that the blue LED peak intensity around 450 nm varies with test time. However, it is known that blue LED chip fabrication is a mature technology and does not degrade easily especially under normal ambient conditions as performed in this test^16^.

**Figure 4:**
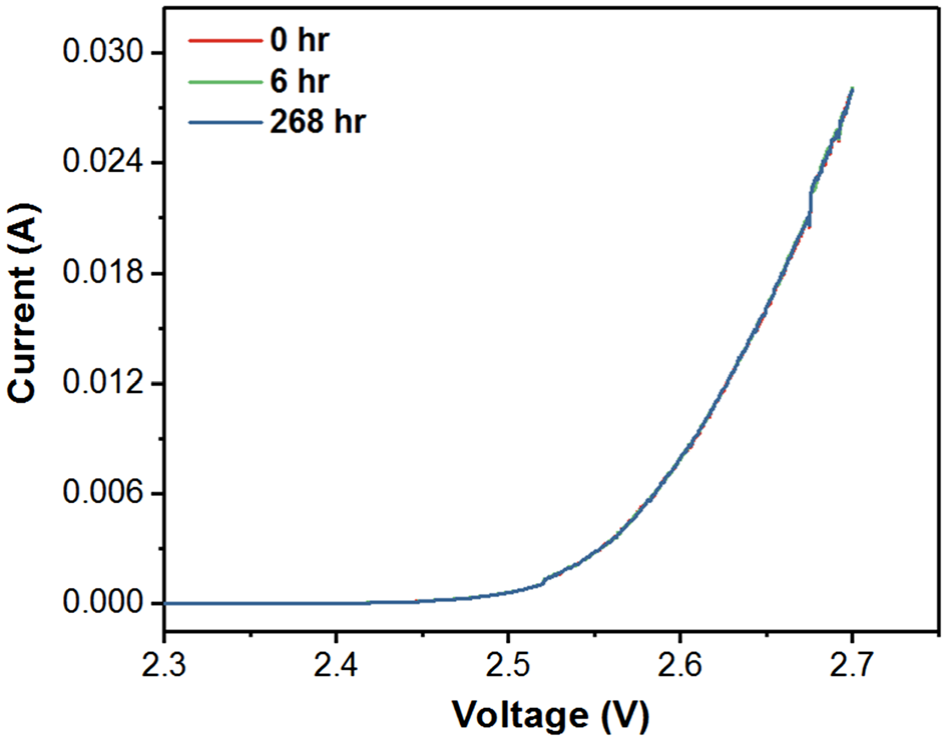
I-V characteristics of LEDs tested under ambient conditions at different time intervals.

To verify the health of the blue LED chips, I-V curves of the blue LED chips is measured with a typical curve shown in Fig. 4. No shift in the I-V curve is observed when tested up to 268 h, implying that the LED chips are intact and do not undergo any degradation, as expected. Therefore, the blue peak intensity variation can be attributed to two factors. First, the loss of blue light may be due to the light scattering caused by moisture that has penetrated inside the encapsulant in the early hours of the test in the similar way as reported in literature^17^. However, in view of the 44% RH in the operating condition at room temperature, such diffusion of moisture into silicone encapsulation is unlikely in this short initial period. Therefore, the blue light intensity variation is likely to be due to the changes in the structure of phosphors or Zn doped CdS/ZnS QDs which can increase the transmittance or absorption of blue light.

**Figure 5:**
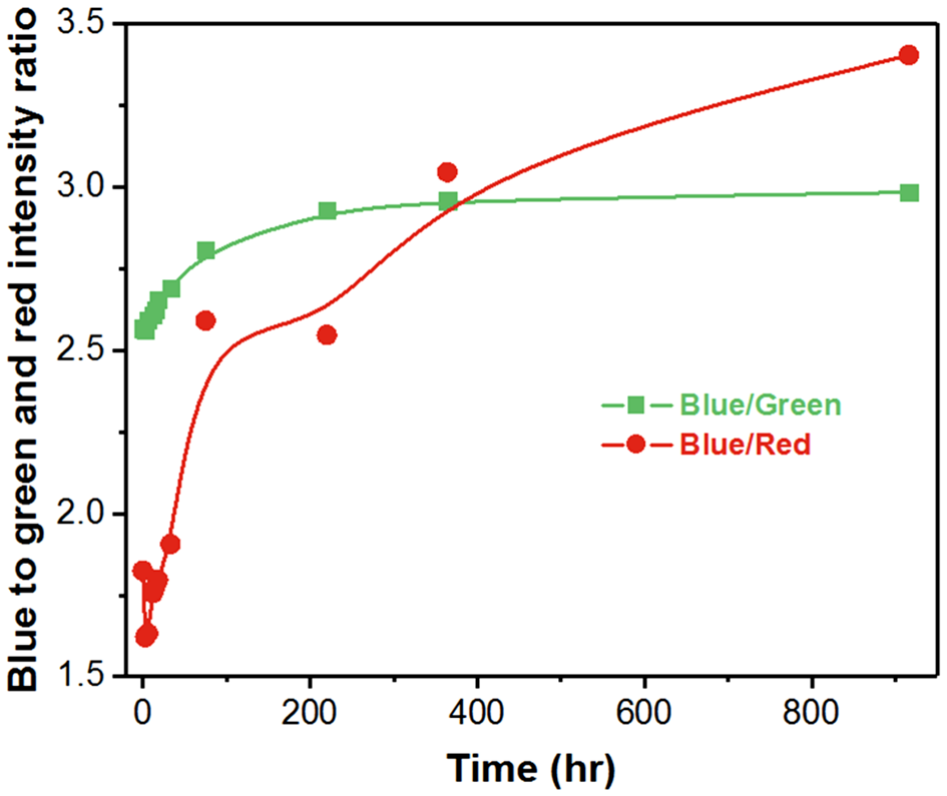
Comparison of light conversion efficiency for phosphor and QD at different time intervals in a packaged LED.

**Figure 6:**
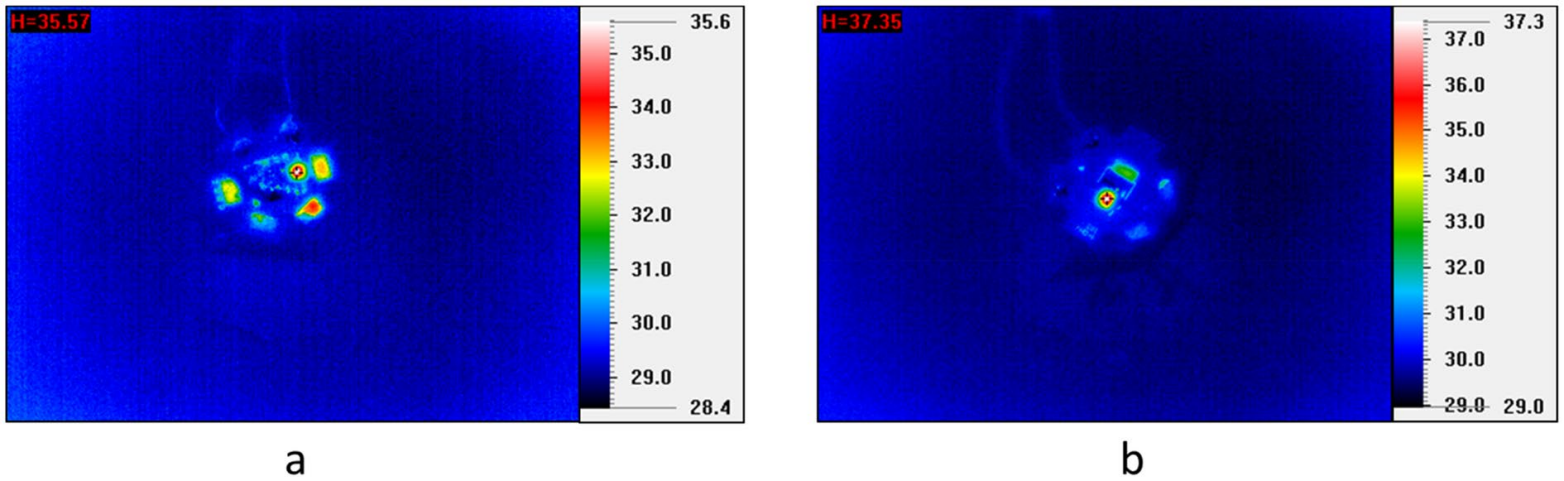
Comparison comparison between heat generation in LEDs (**a**) before and (**b**) after the test. Measurement is done using IR Camera (CHCT P384A-20 Thermal Scanning System).

According to the conversion efficiency plots which are computed as the ratio of blue light intensity and light intensity from phosphor or QDs as shown in Fig. 5, and the blue light is used as a reference since the blue LED chip is intact, the conversion efficiency of QDs and phosphor vary differently, and consequently the variation in the output light intensity vary differently in different stages. It can be observed that the light intensity of phosphor decreases slightly during the first 200 h and then reaches a steady state. The 13% decreases in the phosphor conversion efficiency observed might be attributed to the slight increase of temperature and electro-thermal stress^18,19^, and Fig. 6 indeed shows a rise in temperature for LED before and after the test, with the temperature increased from 35.6 to 37.4 °C and remain constant subsequently. Let us now focus on the degradation of QDs.

### Characteristic of the QDs with operation time

In the enhancement stage, light conversion efficiency of QDs increases during the initial 6 h and followed by a rapid degradation subsequently. The increase in the light intensity from QDs might be attributed to the oxygen adsorption on the surface of QDs which will suppress the inherent surface defect and increase the QDs luminescence as reported by Singh et.al^20^. On the other hand, presence of oxygen atoms can also render photo-oxidation of QD, decreasing the QDs luminescence^21^. Therefore, a competition between passivation of QDs surface defects and photo-oxidation exist, and this could lead to an increase in QDs light output intensity at the first 6th hours followed by the subsequent decrease till 18th hours.

**Figure 7:**
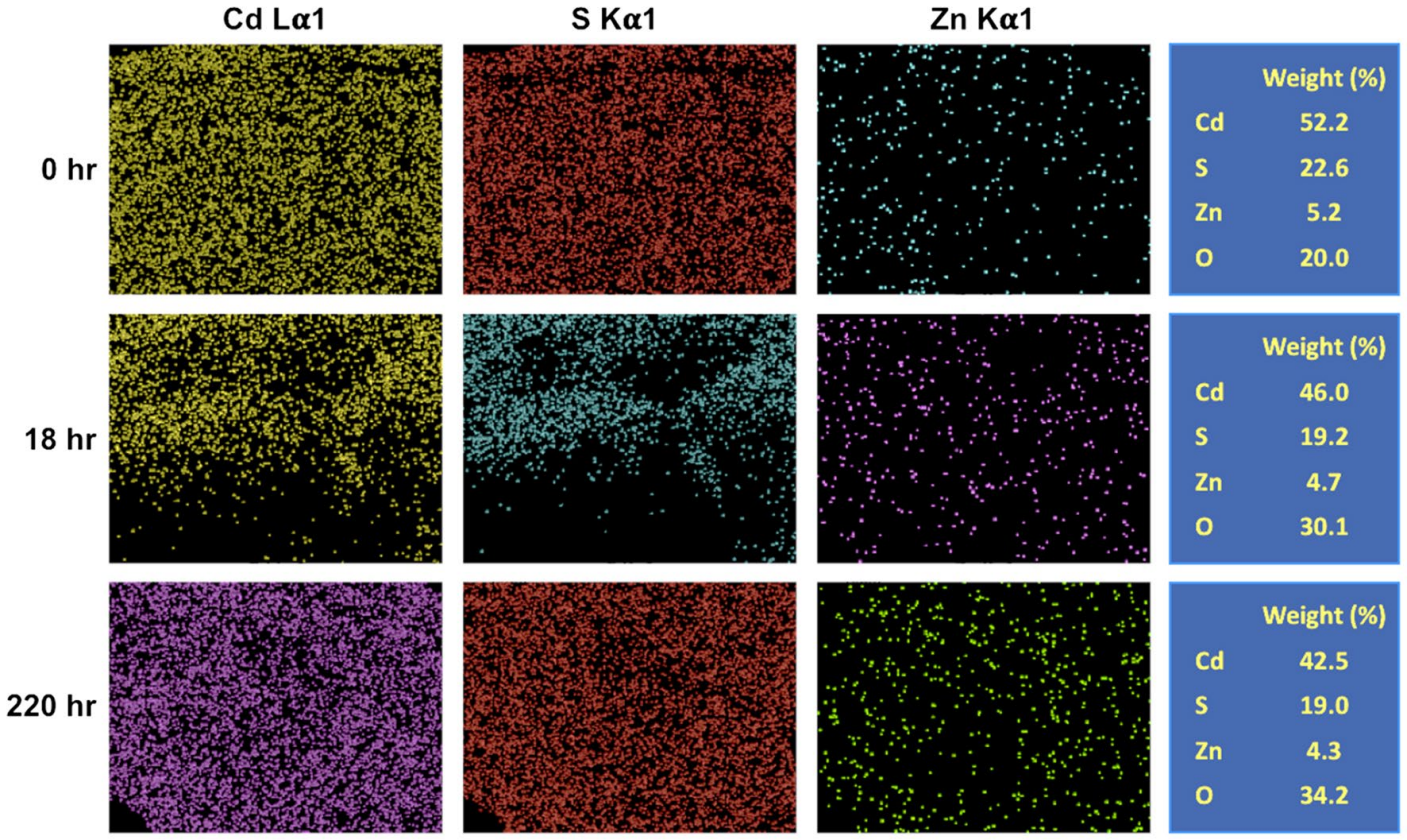
The EDS mapping and element composition of QDs versus test time tested under ambient condition. (Accelerating voltage = 10 kV, working distance = 5.7 mm).

**Figure 8:**
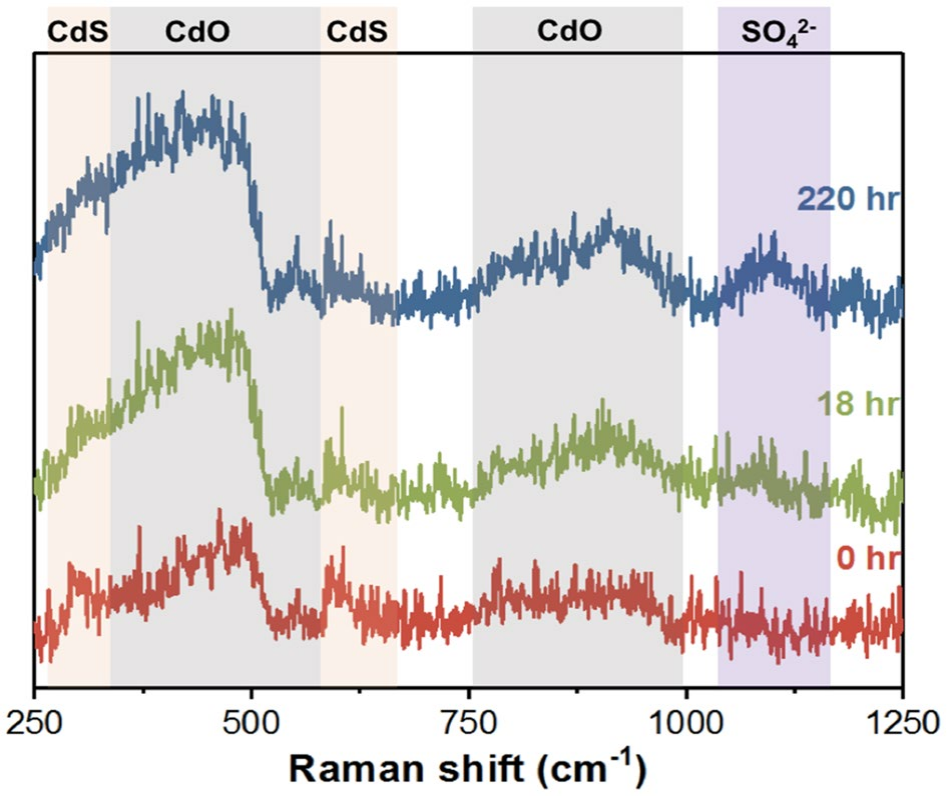
Raman spectra of QDs *vs.* test time tested under ambient condition.

EDS mapping taken using JEOL-JSM SEM/OXFORD EDS with SEI mode is used to examine the composition ratio of elements of the QD, and the results are shown in Fig. 7. Although the shell structure of ZnS is implemented as the protective layer, it can be seen that the distribution of ZnS is not uniform, indicating incomplete surface protection that allow the absorption of oxygen and the oxidation of QD in the presence of light. The photo-oxidation is also confirmed by Raman spectroscopy with the laser wavelength of 785 nm. Two characteristic peaks of the fresh CdS/ZnS QDs are observed at 299 and 597 cm^-1^, which are assigned to the fundamental optical phonon mode (LO) and the first overtone mode (2LO) of CdS^22,23^ (Fig. 8). The characteristic bands of CdO are also found at 529 cm^-1^, broad band at 350 to 500 cm^-1^ and broad band at 750 to 950 cm^-1^ as shown in Fig. 8^24,25^. It is widely known that this defect structure would trap the photo-excited electron and further hinder radiative recombination, thus decreasing the PL intensity of QDs^26,27^. The formation of CdO at S-vacancy reduces the ability of electron trap, resulting in an increase in the PL emission of CdS/ZnS QDs, which is consistent with the result of the enhancement stage. The intensity of CdO peak further increases at 18 h, but the increase in intensity is not obvious at 220 h, implying that major oxidation of CdS to CdO is within the first 18 h. In addition, a new weak band at 1088 cm^-1^ is detected at 18 h, which is assigned to the vibration of the SO_4_^2–^ group of CdSO_4_, and the strength of the band increases with time, indicating that the amount of CdSO_4_ gradually increases during the process^28^.

Comparing the weight ratio of Cd to S element, the values of CdS at 0, 18 and 220 h are 2.31, 2.40 and 2.23, where is an insignificant change. However, there is an increase in the oxygen element content with the test time, which is attributed to the formation of CdO and CdSO_4_ as demonstrated by Raman spectroscopy. To understand the detail of this transformation processes, ab initio DFT based simulation is performed.

### DFT simulation and analysis

**Figure 9:**
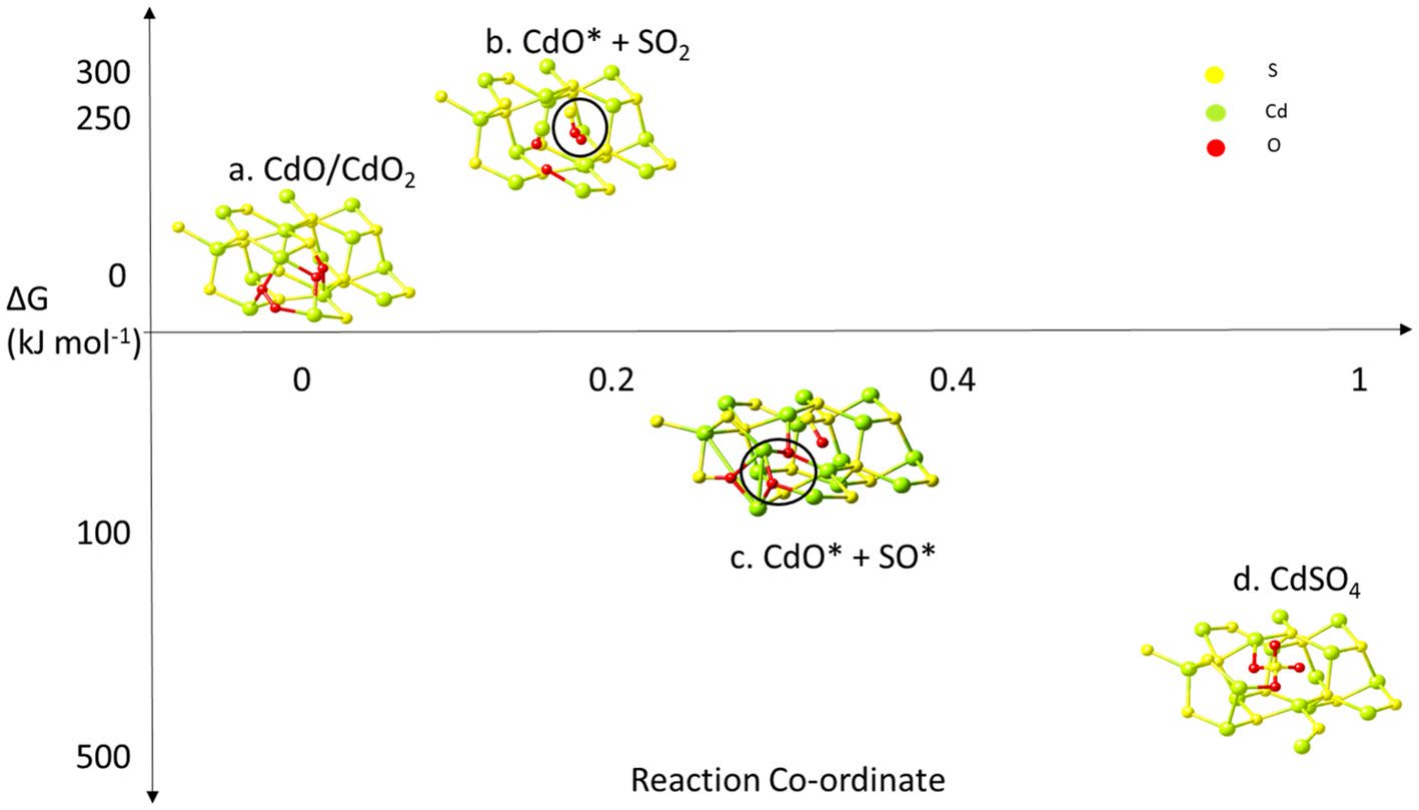
DFT Energy profile of CdO particles oxidizing into CdSO_4_. The black circles indicate the changes taking place in the reaction intermediates.

It is reported that the oxidation of QDs is mainly dominated by oxygen rather than H_2_O^29^, only oxygen is involved in our DFT based simulation. The structure for tracing the Minimum Energy Path (MEP) is designed by assuming that the CdS structure already has a sulphur vacancy resulting in 16 Cd atoms and 15 S atoms in the periodic cell. This sulphur vacancy creates three dangling bonds in the neighboring Cd atoms allowing adsorption of oxygen atoms to form CdO. With this assumption, we introduce two O_2_ molecules near the defect site of the CdS structure. The geometry optimization of the DFT simulation indicates direct adsorption of O_2_ molecules in the defect sites, thereby forming Cd–O/Cd–O_2_ bonds as shown in Fig. 9.

The first intermediate of the degradation process shows the formation of CdO* and SO_2_ molecules in an endergonic process as the ΔG value is positive. This indicates that the formation of this intermediate is non-spontaneous. However, in the presence of an external energy source such as blue light, it is a reachable step because the required energy is 250 kJ mol^-1^ and the energy supplied by the blue light is 254 kJ mol^-1^. However, once the first intermediate is attained, it is quickly dissociated into other intermediates. The transition state of the dissociation reaction is exergonic and more spontaneous than the previous intermediate as the ΔG value is negative.

The transition state indicates dissociation of an O-atom from the SO_2_ molecule, thus creating a SO* complex. The formation of SO_2_ and SO* complex induce further S-vacancy in the CdS structure which was seen to be passivated by the O^*^ ion, forming CdO as shown in Fig. 9b–c. Due to the additional defects induced by the SO_2_ formation, more Cd dangling bonds are created in the structure. These vacant sites are passivated by oxygen with the co-ordination number of O-atom in the CdO complex varied between 3 and 4 as shown in Fig. 9c. However, the co-ordination number of Cd atoms near the defect site changed from 4 to 5 which characterizes the transition state structure as the co-ordination number for Cd(II) atoms are 4 while for Cd(IV) atoms is 6. The bond length of the Cd–O bond length was consistent at 2.27 Å, while the Cd-Cd bond formed in the transition state structure has a bond length of 3.24 Å.

Even though this is a spontaneous intermediate, the atomic arrangement is unstable and this leads to the final stage of the degradation via bond rearrangement. The formation of a CdSO_4_ complex in the CdS nanostructure by oxidation is also an exergonic reaction with a highly negative ΔG. The spontaneity of this reaction complex indicates formation of CdSO_4_ due to sulphur vacancies in presence of O_2_ is a favorable reaction.

Our simulation confirms the constant sulfur concentration as the intermediate of SO_2_ would quickly transform into CdO*, SO* and CdSO_4_ that prevents the loss of sulphur element, which agrees with the constant weight ratio of Cd element to S element from our EDS mapping. On the other hand, the incorporation of oxygen element in the CdS structure to form CdO and CdSO_4_, causes an increase in the oxygen element as observed in our EDS map.

### Photoluminescent spectroscopy analysis of QDs with operation time

**Figure 10:**
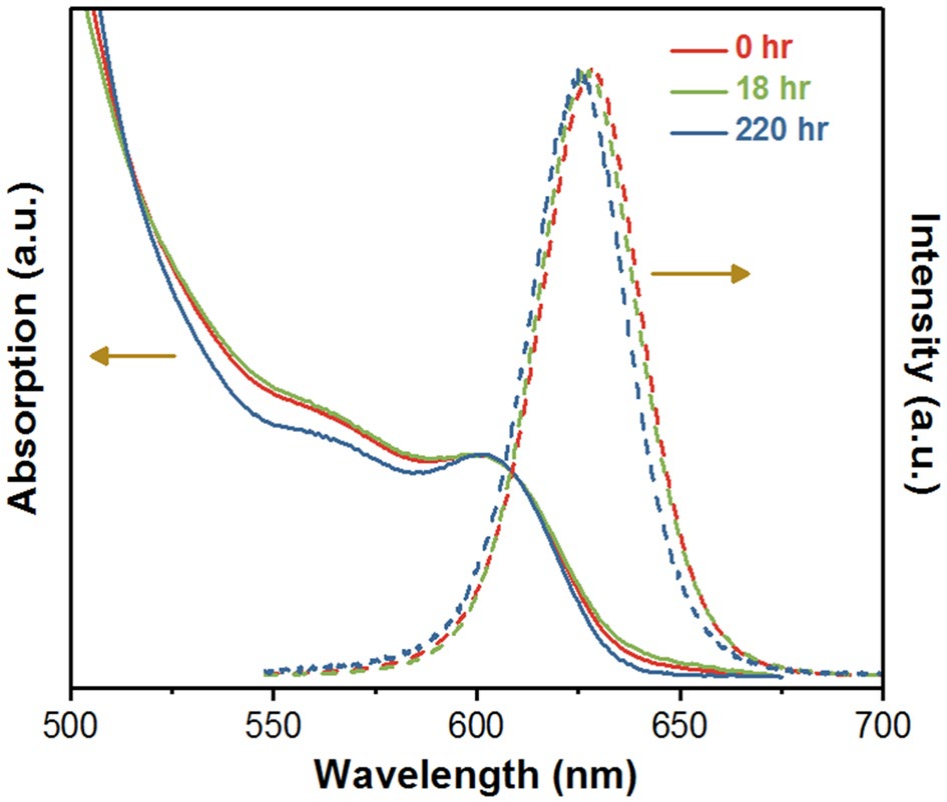
The absorption and PL spectra of QDs *vs.* test time tested under ambient condition.

Figure 10 shows the variations of absorption and PL spectra of CdS/ZnS QDs with time. In the case of QDs at 18 and 220 h of working conditions, the absorption behavior between 500 and 580 nm are different from the fresh QDs. The quantum confinement produces complex structure of hole quantized states due to the mixing valences sub-bands, results in the formation of various interband optical transitions involved in 1S or 1P electron state, thus exhibiting a broad absorption band in the range of 500–580 nm^30^. It means that the difference of absorption behavior within 500–580 nm is attributed to the change of the electronic structure of CdS involved in interband optical transitions due to formation of CdO-CdS, CdSO_4_-CdS and intermediate species which are also predicted in our DFT simulation and demonstrated by Raman spectroscopy. The absorption spectrum of the fresh CdS/ZnS QDs shows a major peak at 602 nm which is the dominating excitonic transitions from ground electronic sub-band to the ground hole sub-band^31,32^. It shows a blue shift to 600 nm and 597 nm at 18 and 220 h, resulting in the blue shift of the emission peak from 628 to 627 nm and 625 nm, respectively. The results of the blue shift of PL emission reveals the decrease of particle size due to the photo-oxidation, which is well in agreement with the quantum size effect and the result of the packaged LED^33^.

**Figure 11:**
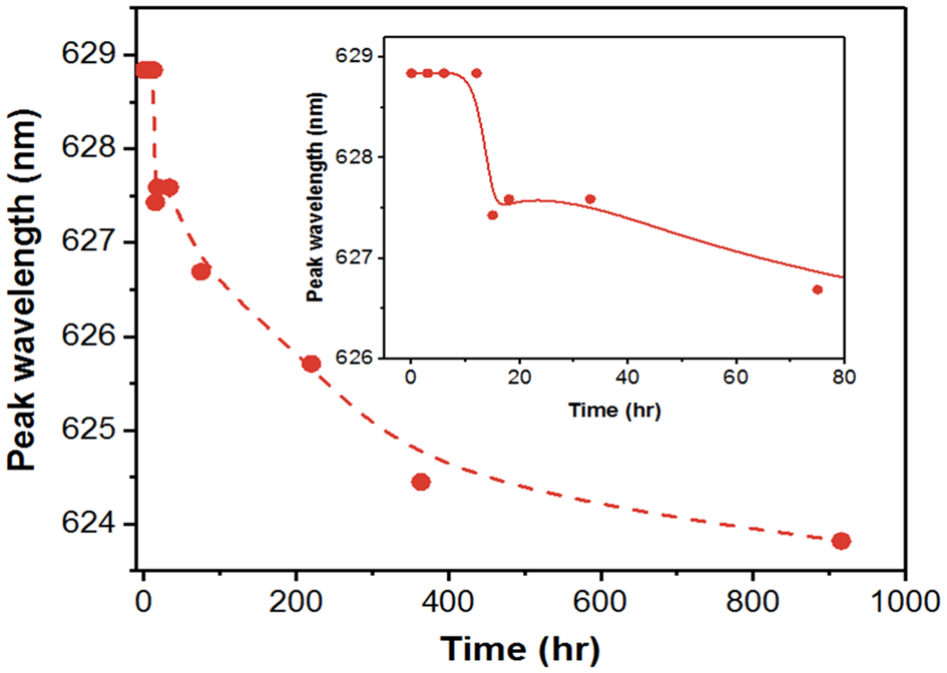
Average of variation of QDs peak wavelength *vs.* test time tested under ambient condition.

It is also observed that a rapid luminescence degradation of QDs is accompanied by a continuous blue shift in QDs peak wavelength from 18 to 364 h of test (Fig. 11). This stage can be considered as an extension of the end of the first stage where photo-oxidation mechanism becomes dominating over surface defect passivation process for QDs because the blue to red intensity ratio in this stage is higher than that in enhancement stage and that the intensity of the red light decreases. This can also be inferred from a previous work on the photo-oxidation of CdSe QDs produced CdSeO_x_ with the decrease of PL intensity, reduction of particle size and blue shift of PL emission^21^, and the formation of CdSO_4_ is also the final product of our simulation. In other words, the reduction of QDs luminescence and the blue shift of QDs emission in rapid degradation stage is attributed to the effect of photo-oxidation.

In the final stage, a negligible overall LED light output change is observed with corresponding slight changes in the QDs luminescence. It seems that the surface oxidation of QDs that resulted in a rapid decrease in wavelength during second stage is slowing down as it enters into this stage. This phenomenon is generally observed in the oxidation of QDs, where the outmost layer of QDs is used for sacrificial oxidation to form a protective oxide layer^34^. This oxide layer could effectively prevent further oxidation of interior QDs. As shown in Fig. 5, the variation of blue to red intensity ratio tends to stabilize, suggesting the surface oxidation of the QDs is nearly complete. The existence of the shell layer can effectively slow down the deep oxidation of QDs^35^.

## Conclusions

White light packaged LEDs are fabricated with the hybrid structure of blue LED, phosphor and QDs in this study. The result of simulation and spectroscopies clearly indicate the impact of oxygen atoms on the durability of Zn doped CdS/ZnS QDs in the presence of blue light which transforms the CdS structure into CdO-CdS and CdSO_4_-CdS structure. This transformation causes lumen degradation and color shift. Our results show that the defect structures in the ZnS protective coating and CdS QD are the starting positions of QDs oxidation. While oxygen atoms are hard to eliminate, the intensity of blue light and the defects of the protective coating over QDs are therefore vital to the durability of QDs in white light LED applications.

## Supplementary Information

Supplementary Information.
