# Supplementary material for: Degradation dynamics of quantum dots in white LED applications
Source: Sci Rep. 2021 Dec 17;11:24153. doi: 10.1038/s41598-021-02714-0 (PMC8683471; doi:10.1038/s41598-021-02714-0)
Supplement: Supplementary file 1 — Supplementary Information. [file 41598_2021_2714_MOESM1_ESM.docx]

**Supplementary information**

**Degradation Dynamics of Quantum Dots in White LED Applications**

*Hsiao-Chien Chen^1,2^, Abdul Shabir^1,3^, Cher Ming Tan^1,3,4,5^*, Preetpal Singh^1^, Jia-Hung Lin^1,3^*

*^1^ Center for Reliability Sciences and Technologies, Chang Gung University, Taoyuan 333, Taiwan*

*^2^ Kidney Research Center, Department of Nephrology, Linkou Chang Gung Memorial Hospital, Taoyuan 333, Taiwan*

*^3^ Department of Electronic Engineering, College of Engineering, Chang Gung University, Taoyuan 333, Taiwan*

*^4^ Urology Department, Chang Gung Memorial Hospital, Taoyuan 333, Taiwan*

*^5^ Center for Reliability Engineering, Ming Chi University of Technology, New Taipei City 243, Taiwan*

^*^ *Corresponding author:* [*cmtan@cgu.edu.tw*](mailto:cmtan@cgu.edu.tw)

*S.1 Preparation of CdS/ZnS core-shell quantum dots (QDs) LEDs*

The CdS/ZnS core-shell QDs was synthesized by hot-injection process. The precursor of Cd was prepared by mixing CdO, oleic acid and octadecene (ODE) at 100^o^C under pumping down condition for 30 mins. Then the temperature was increased to 180^o^C without pumping down to dissolve the CdO completely. The sulfur precursor was prepared by mixing sulfur powder and ODE at 100^o^C under pumping down condition for 30 mins. Then the temperature was increased to 120^o^C without pumping down to dissolve the sulfur powder completely. The precursor of Zn was prepared by mixing zinc octadecenoate and ODE at 100^o^C under pumping down condition for 30 mins before storing at room temperature under N_2_ environment. In the formation of the QD, the temperature of Cd precursor solution was increased to 170^o^C and sulfur precursor was injecting into the reactor slowly. Thereafter, the Zn precursor solution was injected into the reactor rapidly while keeping the temperature for 30 mins. After purifying using chloroform and ethanol, the CdS/ZnS core-shell QDs solution dispersing in benzene could be obtained. The phosphors (yttrium aluminum garnet, YAG) with green light emission was purchased from Grirem Advanced Materials Co., Ltd.

For the fabrication of white-light LED, the mixture latex of phosphors and silicone 6550B were directly dispensed on an InGaN/GaN blue LED chip purchased from EPISTAR Corporation. To obtained evenly dispersed CdS/ZnS QDs latex, the QDs benzene solution was mixed with silicone 6650B and they were placed in a degassing system for 30 mins. The well dispersed QDs solution then dispensed on phosphors layer and kept at 80^o^C in an oven for 30 mins followed by curing at 120^o^C for 2 hours. The final product was a white LED. All the LEDs performed in this work were prepared in the exact same way.

*S.2 Atomic scale modelling and simulation methodology*

In order to understanding the detail degradation mechanisms as observed experimentally, atomic scale modeling is employed. It has been established that CdS nanostructures have a dominant hexagonal crystal structure^[1,2]^. With this reference, we used a P63mc space group CdS structure from Medea’s Infomatica database^[3]^ and extended it into a 2 x 2 x 2 supercell consisting of 16 Cd and S atoms respectively.

The density functional theory (DFT) calculations are carried out by projector-augmented wave (PAW) method^[4]^ as implemented in the Vienna Ab-initio Simulation Package provided by Medea (Medea-VASP)^[3,5]^. The interactions are described using the Generalized Gradient Approximation with Perdew-Burke-Ernzerhof (GGA-PBE) exchange-correlation functional^[6]^. The atomic orbitals are defined by plane-wave basis sets with cut-off energies of 300 eV^[7]^. Reciprocal space projection operators are used and the convergence criterion for self-consistent field calculations is set to 10^-5^ eV between consecutive steps. The convergence criterion for atomic forces of the systems is set to be less than 0.002 eV A^-1^. The Brillouin zone is sampled at 2 x 2 x 1 gamma centered k-points where the actual k-point spacing is 0.439 x 0.439 x 0.465 per Angstrom.

The transition state search (TSS) module of the MedeA software is used to observe the energy changes between the initial and final structures^[3,8]^. In the TSS module, we employ Nudged elastic band (NEB) method^[8,9]^ to map the minimum energy path (MEP) between the initial and the final systems by calculating the free energy (ΔG) barriers at different reaction stages. The stage closest to a saddle point is allowed to climb up into the saddle point (cNEB method) if the largest force on an atom is smaller than 1.0 eV Å^−1^. The Self Consistent Field (SCF) is calculated by RMM_DIIS minimizer method^[7,10]^. Elements of the inverse Hessian are initially set to 0.001 Å^2^ eV^−1^and linear interpolation is used to create the reaction intermediates. Reaction coordinate is the normalized coefficient of linear interpolation with translation criterion of 0.5.

*S.3 Equipment and measurements*

The structural information of CdS/ZnS QDs with time was analyzed using Raman spectroscopy UniNano UNIDRON with a Raman microscopy system, employing a diode laser at 785 nm. A 50× objective lens was used to focus the laser on the sample, in which the size of the laser spot is 1 μm. Meanwhile the Raman spectrum measurement was performed under an exposure time of 2 s and an accumulation number of 30 times by illuminating 600 mW of laser power. The measurement of PL spectroscopy was obtained using UniNano UNIDRON with a PL microscopy system. The excited wavelength was selected at 532 nm with an exposure time of 1 s and an accumulation number of 5 times. Additionally, absorption spectra were recorded on a JASCO V–670 spectrometer.

**References**

[1] M. Micheel, B. Liu, M. Wächtler, *Catalysts* **2020**, *10*, 1.

[2] Z. Chen, P. Z. Zhang, Y. Zhou, X. Zhang, X. Liu, Z. Hou, J. Tang, W. Li, *J. Phys. Chem. Lett.* **2020**, *11*, 10354.

[3] G. Socrates, Infrared and Raman Characteristic Group Frequencies: Tables and Charts, 3rd Edition. **2004**

[4] C. W. Yeh, G. H. Chen, S. J. Ho, H. S. Chen, *ACS Appl. Nano Mater.* **2019**, *2*, 5290.

[5] V. I. Klimov, *Annu. Rev. Phys. Chem.* **2007**, *58*, 635.

[6] D. U. Lee, D. H. Kim, D. H. Choi, S. W. Kim, H. S. Lee, K.-H. Yoo, T. W. Kim, *Opt. Express* **2016**, *24*, A350.

[7] Y. Wang, V. D. Ta, Y. Gao, T. C. He, R. Chen, E. Mutlugun, H. V. Demir, H. D. Sun, *Adv. Mater.* **2014**, *26*, 2954.

[8] W. William Yu, X. Peng, *Formation of High-Quality CdS and Other II ± VI Semiconductor Nanocrystals in Noncoordinating Solvents: Tunable Reactivity of Monomers***, **2002**.

[9] L. Huang, Z. Li, C. Zhang, L. Kong, B. Wang, S. Huang, V. Sharma, H. Ma, Q. Yuan, Y. Liu, G. Shen, K. Wu, L. Li, *Chem. Sci.* **2019**, *10*, 6683.

[10] V. John, in *Introd. to Eng. Mater.*, Palgrave Macmillan UK, **1992**, pp. 419–433.
